# Natural Marine and Terrestrial Compounds as Modulators of Matrix Metalloproteinases-2 (MMP-2) and MMP-9 in Alzheimer’s Disease

**DOI:** 10.3390/ph14020086

**Published:** 2021-01-24

**Authors:** Lidia Ciccone, Jennifer Vandooren, Susanna Nencetti, Elisabetta Orlandini

**Affiliations:** 1 Department of Pharmacy, University of Pisa, via Bonanno 6, 56126 Pisa, Italy; lidia.ciccone@unipi.it (L.C.); susanna.nencetti@unipi.it (S.N.); 2 Laboratory of Immunobiology, Department of Microbiology, Immunology and Transplantation, Rega Institute for Medical Research, University of Leuven, KU Leuven—Herestraat 49—Box 1044, 3000 Leuven, Belgium; jennifer.vandooren@kuleuven.be; 3 Interdepartmental Research Centre “Nutraceuticals and Food for Health (NUTRAFOOD), University of Pisa, 56126 Pisa, Italy; 4 Department of Earth Sciences, University of Pisa, via Santa Maria 53, 56126 Pisa, Italy; 5 Research Center “E. Piaggio”, University of Pisa, 56122 Pisa, Italy

**Keywords:** MMP-2, MMP-9, Alzheimer’s disease, AD, neurodegeneration, neuroinflammation, natural compounds, marine compounds, terrestrial compounds, nutraceuticals

## Abstract

Several studies have reported neuroprotective effects by natural products. A wide range of natural compounds have been investigated, and some of these may play a beneficial role in Alzheimer’s disease (AD) progression. Matrix metalloproteinases (MMPs), a family of zinc-dependent endopeptidases, have been implicated in AD. In particular, MMP-2 and MMP-9 are able to trigger several neuroinflammatory and neurodegenerative pathways. In this review, we summarize and discuss existing literature on natural marine and terrestrial compounds, as well as their ability to modulate MMP-2 and MMP-9, and we evaluate their potential as therapeutic compounds for neurodegenerative and neuroinflammatory diseases, with a focus on Alzheimer’s disease.

## 1. Introduction

Most neurodegenerative diseases are characterized by an incurable loss of neurons in the brain and spinal cord, leading to impaired movement and/or mental functioning. In 2019, an estimated 50 million individuals were suffering from dementia worldwide, and this number is projected to increase to 152 million cases by 2050. The worldwide cost of dementia exceeds one trillion dollars per year and is set to double by 2030 [1]. While Alzheimer’s disease (AD) is the most common neurodegenerative disorder, other neurodegenerative disorders resulting in cognitive defects include vascular dementia, frontotemporal dementia, mixed dementia, and dementia with Lewy bodies. Furthermore, neurodegenerative diseases affecting the motor system include amyotrophic lateral sclerosis (ALS), Huntington’s disease (HD), Parkinson’s disease (PD), and spinocerebellar ataxias [2]. Despite the high burden of these pathologies, current therapies mostly manage symptoms but do not prevent progressive deterioration [3,4].

**Figure 1:**
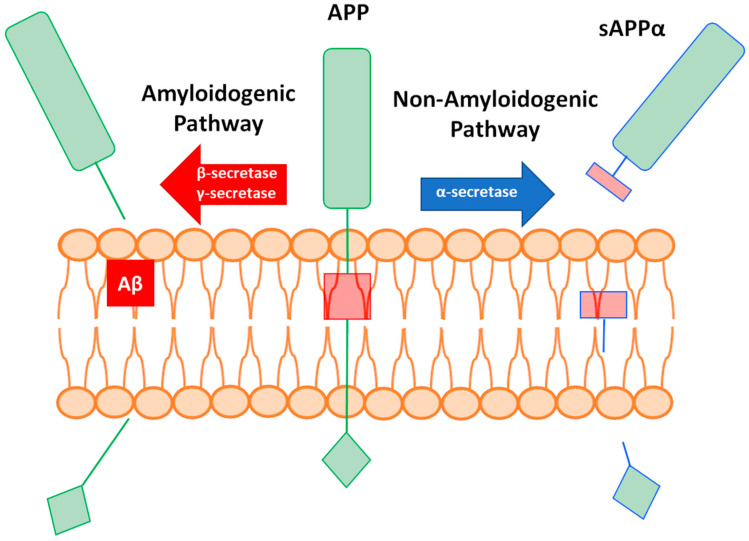
Graphical representation of amyloid precursor protein (APP) cleavage and the amyloidogenic and non-amyloidogenic pathways. sAPPα: soluble APPα.

Brain tissue from patients with AD is characterized by the presence of extracellular amyloid-β (Aβ) plaques and intracellular neurofibrillary tangles of hyperphosphorylated tau proteins [5]. The most known molecular pathway in AD is the generation of Aβ peptides, which are released after the consecutive cleavage of membrane-associated amyloid precursor protein (APP) by α and γ-secretases (a process referred to as the amyloidogenic pathway; Figure 1). After release, Aβ peptides induce the formation of protein aggregates. While the main component is Aβ, up to 488 other proteins that also influence the process of aggregation have been detected [6]. For example, a cross-reaction with apolipoprotein A1, cystatin C, or transthyretin inhibits amyloid formation [7,8,9,10,11,12]. However, the formation of pathogenic Aβ peptides can be avoided through the cleavage of APP by α-secretase and the release of soluble APPα (sAPPα) (a process referred to as the non-amyloidogenic pathway; Figure 1) [13]. Though tau and Aβ aggregates are hallmarks of AD [14], the failure of clinical trials targeting Aβ (resulting in adverse effects on cognition) has sparked a debate regarding whether the production of Aβ is the primary underlying cause of AD [15].

One family of proteins that has gained attention in neurodegeneration and neuroinflammation are the matrix metalloproteinases (MMPs). MMPs are endopeptidases that are able to modify a broad range of proteins with key functions in the extracellular and pericellular environment (discussed in Section 1.1.). MMPs have been studied as therapeutic targets for several pathologies, in particular cancer. However, early clinical trials in which broad spectrum metalloproteinase inhibitors were used for the treatment of cancer failed due to off-target effects. Since then, selectivity has become one of the principal aspects of MMP inhibitor design. In addition, various molecules acting against two or more MMPs have recently been investigated, suggesting that carefully selected multitarget approaches also represent a promising strategy for targeting MMPs [16].

Interesting, both the beneficial and detrimental functions of MMPs in neurodegeneration and neuroinflammation have been described [17]. For example, MMPs cleave myelin basic protein (MBP), thereby contributing to the demyelination of neurons and neurodegeneration. In contrast, several MMPs are able to degrade Aβ aggregates [18,19]. Though this suggests a potential role for MMPs in aggregate clearance, it remains to be seen whether this is clinically significant. Nevertheless, given the many pathological functions of MMPs, they remain considered as potential targets for neurodegenerative diseases such as AD. An overview of the roles of MMPs in neuropathology and discussion on their suitability as targets in AD is given in Section 1.2.2.

Better knowledge on products that modulate MMPs could inspire new approaches for treatment of neurodegenerative and neuroinflammatory diseases such as AD. Natural bioactive products extracted from plants, minerals, animals, and microorganism are common claimed nutraceuticals, and they have been used to fight many diseases [20]. Knowledge on medicinal use of natural products is often handed over from one generation to another, and it stems from thousands of years ago when medicinal plants, rich in phytochemicals and microorganisms, were a major source of medicines.

Recently, many natural products were placed under investigation in pre-clinical and clinical trials in the treatment of AD [21], confirming the relevance of natural compounds. Several studies report that nutraceuticals and phytochemicals can have a crucial role in cell survival, neuron function, synaptic plasticity, and memory formation, thus contributing to prevention of neurodegenerative diseases onset [22,23,24]. Furthermore, in addition to other functions such as antioxidant activity and anti-inflammatory activity, several of these compounds are able to modulate the expression or proteolytic activity of MMPs.

In this manuscript, we briefly discuss MMPs and their known roles in neurodegeneration and neuroinflammation, followed by an overview of natural compounds derived from marine and terrestrial sources that can modulate the expressions level and/or activity of MMP-2 and MMP-9. The aim of this manuscript is to function as a reference for medicinal chemists who wish to develop new molecules that combine the beneficial actions of natural compounds with the ability to modulate MMPs, perhaps being useful in the treatment of neuropathologies such as AD.

### 1.1. Introduction to MMPs

**Figure 2:**
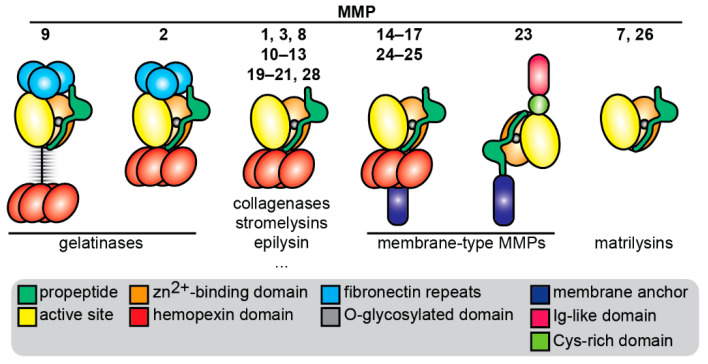
Overview of matrix metalloproteinases (MMPs) and their functional domain organization. Cys: cysteine; Ig: immunoglobulin.

Matrix metalloproteinases (MMPs), also called matrixins, are a family of zinc-dependent endopeptidases [25]. MMPs have a modular domain structure and share a similar catalytic domain and zinc-binding domain, together forming the active site for substrate catalysis (Figure 2). MMPs are secreted by cells such as inactive pro-enzymes (proMMPs) that require the proteolytic removal (e.g., by other proteases) or chemical modification (e.g., by reactive oxygen species) of a self-inhibitory prodomain in order to become catalytically active proteases [26]. The prodomain, catalytic domain, and zinc-binding domain form the structural basis of all MMPs. Several MMPs also share a hemopexin domain that is involved in binding to substrates [27], inhibitors [28], and cell surface receptors [29]. MMP-2 and MMP-9 (gelatinase A and gelatinase B) both have three fibronectin repeats that give them the ability to bind large substrates such as collagens and to efficiently cleave gelatins [30]. In addition, MMP-9 has a unique linker sequence (64 amino acids) that connects the active site to the hemopexin domain and is rich in O-glycans; hence, it is named the O-glycosylated domain. This domain is indispensable for MMP-9 functions including cell migration [31], substrate catalysis [32], and the regulation of bioavailability [33]. Furthermore, this domain allows MMP-9 to form higher order multimers that are differentially regulated by natural MMP inhibitors than MMP-9 monomers [34]. There are several MMPs that are bound to the cell surface through a membrane anchoring domain such as a transmembrane domain or glycosylphosphatidylinositol anchor, thereby executing cell-surface associated functions [25]. Finally, MMP-23 is an atypical MMP, having a transmembrane domain attached to the N-terminus and an immunoglobulin-like domain and cysteine-rich domain attached to the C-terminus [35].

MMPs are mainly known for their ability to cleave components of the extracellular matrix (ECM); hence, their original nomenclature was based on substrate specificity, e.g., collagenases, gelatinases, and matrilysins. The cleavage of ECM components by MMPs contributes to processes such as tissue remodeling, cell migration, and the release of growth factors from the ECM [36]. However, many other (even intracellular [37]) MMP substrates have been found, and intracellular roles of MMPs are being discovered [38,39]. For example, the nuclear localization of MMP-2 was reported in cigarette smoke-exposed endothelial cells and associated with cell apoptosis [40]. MMP-12 is transported to the nucleus, where it binds to the NF-kappa-B inhibitor alpha NFKBIA promoter and mediates NFKBIA transcription, leading to Interferonα secretion and protection against viral infections [41]. Finally, functions unrelated to the proteolytic actions of MMPs have also been found. These effects mainly rely on the cell-surface association of MMPs and the subsequent activation of signal transduction pathways, e.g., by binding of cell surface receptors such as CD44 and integrins [42].

### 1.2. MMPs in Neurodegeneration and Neuroinflammation

A common feature of several neurodegenerative disorders is the presence and dispersal of anomalous protein aggregates throughout the brain. For example, the formation of amyloid fibrils and Aβ peptide aggregates in AD, tau tangles in tauopathies and α-synuclein aggregates in PD. The formation of these anomalies is associated with neurotoxicity and the progressive loss of neurons. As an additional factor, innate immune mechanisms are also implicated in the pathogenesis of neurodegenerative diseases. The transitioning of the innate immune response into chronic inflammation results in the proliferation of glial cells (gliosis) and elevated levels of proinflammatory cytokines [2]. For a comprehensive overview of the actions of all MMPs in neurodegeneration and neuroinflammation, we refer the reader to several recent reviews [17,43,44,45]. In this manuscript, we focus on the actions of the two gelatinases—MMP-2 and MMP-9. 

#### 1.2.1. Localization and Origin of MMP-2 and MMP-9 in the Nervous System

Both MMP-2 and MMP-9 can be expressed by cells of the nervous system. While expression of MMP-9 is most often induced, MMP-2 is more constitutively present and less influenced by damaging factors [46]. The basal expression of MMP-9 in healthy brain tissue is low [47] but increases significantly in disease models and/or patient samples with neurological damage. The disease- or damage-mediated induction of MMP-9 is found in the endothelial cells of cerebral vasculature [47], astrocytes surrounding amyloid plaques or in injured nerves [18,48,49], areas of astrogliosis [50], meninges and neurons of the injured spinal cord [51], human pyramidal neurons [52], and Schwann cells stimulated with proinflammatory cytokines (e.g., tumor necrosis factor-α and interleukin-1β) [53]. In patients with secondary progressive multiple sclerosis (MS), MMP-9 is expressed at the rim of plaques in chronic active lesions, suggestive for the expression of MMP-9 by activated microglia [54]. Furthermore, all forms of MMP-9 (monomers, multimers, and charge variants) are increased in serum from patients with MS [55]. Interestingly, in a rat model for HD, MMP-9 immunoreactivity was found in the nuclei of the neurons of a healthy rat striatum [50]. In a mouse model for peripheral nerve injury, MMP-2 was found to be constitutively present in nerve tissue [56]. However, in a mouse model for AD, MMP-2 was also increased in astrocytes surrounding amyloid plaques [18].

Infiltrating immune cells are a second source of MMP-2 and MMP-9. Polymorphonuclear leukocytes (PMNs) are the first cells to arrive in damaged tissue and PMN-derived MMPs have neurotoxic activity [57]. PMN infiltration is followed by monocyte infiltration, and tissue-differentiated monocytes secrete higher levels of MMP-9 compared undifferentiated monocytes, thereby contributing to neurotoxicity [58]. In animal models for peripheral nerve injury and spinal cord injury, MMP-9 is associated with infiltrating macrophages [56,59]. In HIV-induced dementia, proMMP-2 is secreted by HIV-infected macrophages [56], and in a model for neuroinflammation, macrophage-derived gelatinase (MMP-2 and MMP-9) activity was found to be crucial for leukocyte infiltration into the central nervous system (CNS) [60]. In conclusion, there are several origins of MMP-2 and MMP-9 in CNS pathology; both resident and infiltrating immune cells are able to supply these proteases or induce their production, e.g., upon stimulation with pro-inflammatory molecules.

#### 1.2.2. Mechanisms of MMP-2 and MMP-9 in CNS Pathology

**Figure 3:**
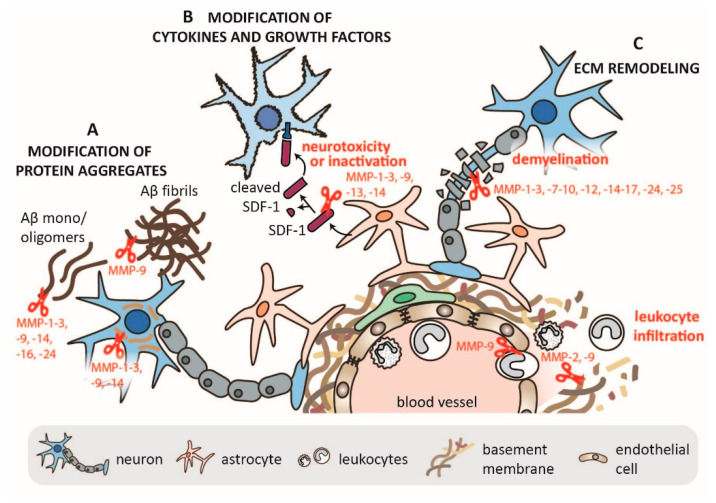
Overview of MMP functions in the nervous system. (**A**) Several MMPs can cleave protein aggregates such as Aβ and tau, with MMP-9 being unique in its ability to cleave Aβ fibrils. (**B**) MMPs can modify several cytokines and growth factors. This is exemplified by the cleavage of stromal cell-derived factor-1 (SDF-1)/ C-X-C motif chemokine Ligand 12 (CXCL12) and either its inactivation or its conversion into a neurotoxic peptide. (**C**) MMPs cleave several components of the extracellular matrix (ECM) such as tight junction proteins and components of the basement membranes, thereby allowing for immune cell migration and contributing to neuroinflammation.

MMPs have a potential role in the turnover of protein aggregates (Figure 3). Several MMPs, including MMP-2 and MMP-9, are able to cleave Aβ monomers and oligomers, but MMP-9 is unique in its ability to also cleave Aβ fibrils and clear plaques from amyloid-laden brains. For a detailed overview of MMP cleavage sites on APP, the reader is referred to the review manuscript by Cauwe et al. [61]. A recent overview of APP processing by MMPs is also available in the review manuscript by Zipfel et al. [16]. Though mainly relying on in vitro studies, ex vivo studies, and steady state mouse models, a positive cross-interaction between Aβ, MMP-2, and MMP-9 has been suggested [18,48]. Moreover, several studies have shown that Aβ can also induce MMP-9 expression and activity in vitro, e.g., in astrocytes [48] and in THP-1 cells (a monocytic cell line) [62]. Hence, Aβ-induced MMP-2 and MMP-9 expression might also enhance MMP-associated neurotoxicity and outweigh the Aβ-clearing effect. Interestingly, tau, the aggregating protein associated with tauopathies, including AD, is also an MMP-2, MMP-3, and MMP-9 substrate. However, proteolysis by MMP-9, not MMP-3, induces tau oligomer formation [63], whereas a physiological function of MMP-2 in normal tau proteolysis has been suggested [64].

MMP-2 and MMP-9 can cleave several soluble factors such as chemokines and growth factors, thereby altering their functional properties [65]. Several MMPs (including MMP-2 and MMP-9) are able to cleave C-X-C motif chemokine Ligand 12 (CXCL12) stromal cell-derived factor-1 (SDF-1), thereby either degrading it or converting it to a neurotoxic protein that activates the intrinsic apoptotic pathway (Figure 3) [66]. In HIV-associated neurodegeneration, proMMP-2 is secreted by HIV-infected macrophages, and after activation by neuronal MMP-14/ membrane-type 1 (MT1)-MMP, MMP-2 converts astrocyte-derived SDF-1 into neurotoxic SDF-1 (5–67) [67]. Another crucial substrate of MMPs is MBP (Figure 3). Many in vitro and in vivo studies have shown that MMPs promote demyelination by degrading MBP, a major constituent of myelin sheets supporting neuronal signals [53,68,69,70].

MMPs also have a fundamental role in leukocyte migration (Figure 3). During neuroinflammation, leukocytes migrate from the circulation into the CNS. This process requires their migration through a layer of vascular endothelial cells, two layers of basement membranes (the endothelial basement membrane and the parenchymal basement membrane), and a layer of astrocytes, together forming the blood brain barrier (BBB) [36]. Both MMP-2 and MMP-9 are important players in this process. In mouse experimental autoimmune encephalomyelitis (EAE), a model for CNS inflammation, leukocytes were found to use MMP-2 and MMP-9 to migrate through the parenchymal basement membrane, specifically through the cleavage of dystroglycan, a transmembrane receptor that connects astrocyte endfeet with parenchymal basement membrane BM [60]. For a detailed overview on the roles of MMP-2 and MMP-9 in neuroinflammation, we refer the reader to a recent review by Hannocks et al. [45].

Several models for neurodegeneration and neuroinflammation in animals deficient in MMP-2 and/or MMP-9 have been used to show beneficial effects on disease progression and outcome. Upon spinal cord injury, MMP-9-knockout (KO) mice have less disruption of the blood–spinal cord barrier, reduced neutrophil infiltration, and improved locomotor recovery [51]. The deletion of the MMP-9 gene protects nerve fibers from demyelination and reduces neuropathic pain after injury [53]. While MMP-9 KO mice are protected in traumatic brain injury [71], in ischemia, the knock-out of MMP-2 does not alter acute brain injury [72]. The genetic ablation of both MMP-2 and MMP-9 in mice results in resistance to EAE by inhibiting dystroglycan cleavage and preventing leukocyte infiltration [60]. Finally, the beneficial effects of MMP inhibitors (minocycline, simvastatin, and GM6001) have been described in a model for cerebral amyloid angiopathy [73]. Overall, these studies justify the inhibition of MMP-2/-9 in neurodegenerative and neuroinflammatory diseases such as AD and highlight the potential of new inhibitory compounds.

## 2. Natural Products from Marine Source That Modulate MMP-2 and/or MMP-9

Marine organisms such as sponges, macroalgae, microalgae, and bacteria are considered effective biological sources of new bioactive drugs. These organisms are usually rich in nutraceuticals and pharmaceuticals that are secreted to survive in the hostile environment where they live. The bioactive molecules are metabolites such as small chemical molecules, as well as short peptides and enzymes. One critical point associated with marine compounds is that only limited amounts are produced by the natural source. In addition, their chemical structures are often too complex to be synthetized in vitro. In recent years, several products with anti-MMP activity have been identified from marine sources. Most investigations have been focused on their effect on MMPs during inflammatory diseases and/or cancer. Due to the relevance of MMPs (especially MMP-2 and MMP-9) in neurodegenerative diseases, marine products could also be useful for the inhibition of MMPs in AD [74].

**Figure 4:**
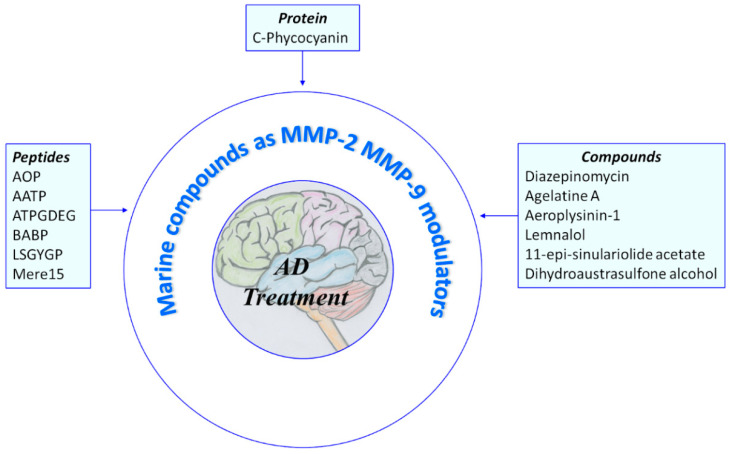
Overview of marine compounds able to modulate MMP-2 and MMP-9. AOP: abalone oligopeptide; AATP: abalone anti-tumor peptide; ATPGDEG: Ala-Thr-Pro-Gly-Asp-Glu-Gly; BABP: boiled abalone byproduct peptide; LSGYGP: Leu-Ser-Gly-Tyr-Gly-Pro; Mere15: Mere Meretrix 15 kDa polypeptide.

In the following paragraphs, several natural MMP modulators of marine origin are reported. For more detailed information regarding the literature prior to 2018, we refer the reader to previous reviews cited below [75,76,77]. The active products are divided in two groups: protein/peptides and small molecules (Figure 4).

Of note, many manuscripts reporting on the analysis of MMPs (in particular MMP-2 and MMP-9) miss a clear distinction between differences in mRNA levels, protein levels, and proteolytic activity. This problem stems from the fact that several of the standard methods to evaluate MMP-2 and MMP-9 levels and activity are prone to misinterpretation (as discussed in more detail by Vandooren et al., 2013) [78]. In this review manuscript, a clear distinction between these different levels of regulation was made based on the methodology used in each of the discussed manuscripts.

### 2.1. Protein and Peptides

**Figure 5:**
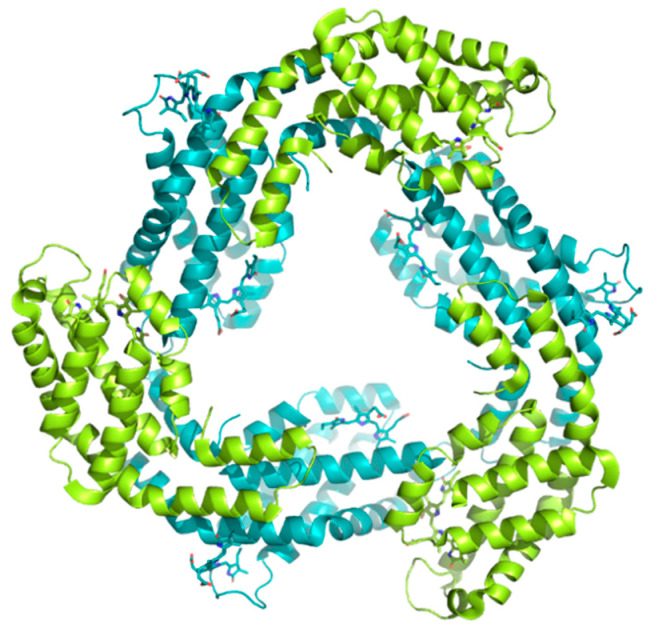
Graphical representation of the C-phycocyanin (C-PC) crystal structure PDB code 3O18 [80]. In this structure, C-PC forms trimers (αβ)_3_. The α subunit is colored in lemon, and the β subunit is colored in teal.

C-phycocyanin (C-PC) is a deep blue colored pigment protein that can be isolated and purified from several seaweeds. C-PC is largely found in *Spirulina*, a microalgae used in many countries as dietary supplement and whose nutritional benefits have been well-described [79]. The structure of C-PC is characterized by a heterodimeric monomer (αβ) formed by the α and β subunits. Usually, the αβ monomers of C-PC further polymerize into higher order multimers; (αβ)_n_ *n* = 1 ~ 6 [80]; see Figure 5.

C-PC has several biological activities such as improving wound healing, antioxidant activity, pro-apoptotic effects, and antitumor activity [81]. The beneficial effects of C-PC have also been observed in various models for degenerative diseases such as PD, MS and ALS [82,83,84].

Recently, C-PC was reported as an inhibitor of MMP-2 and MMP-9. In a vasculogenic mimicry assay with breast cancer cells (MDA-MB 231 cell line), treatment with C-PC resulted in a drastic reduction in the number of vascular channels formed compared to a control. A real-time quantitative reverse transcription-PCR (qPCR) analysis recorded a decrease in the mRNA levels of both vascular endothelial growth factor receptor-2 (VEGFR2) and MMP-9, two key regulators of cancer-associated angiogenesis [85].

In a 1,2-dimethylhydrazine-induced colon cancer rat model, treatment with piroxicam and C-PC resulted in a lower tumor expression of MMP-2 and MMP-9 compared to a control. Both pro- and activated forms of MMP-2 and MMP-9 were reduced [86]. In the HepG2 cell line (hepatocellular carcinoma cell line), C-PC could also inhibit the mRNA and protein expression of both investigated MMPs [87]. Moreover, it has been reported that C-PC might cross the BBB in a model for tributyltin chloride (TBTC)-induced neurotoxicity, adding to its neuroprotective effects and making C-PC a potential drug candidate in neurodegenerative diseases [88].

Abalone, *Haliotis discus hannai*, is a marine univalve gastropod that is predominantly cultured on the southwestern coast of Korea and considered as a precious food at Asian markets. Several studies have found that abalone is a source of nutraceuticals with anti-microbial, anti-oxidant, anti-thrombotic, anti-inflammatory, and anti-cancer activities [89].

The digestion of abalone intestine with an in vitro gastrointestinal (GI) digestion system resulted in the identification of two peptides with anti-MMP-2 and anti-MMP-9 activity in human fibrosarcoma cells (HT1080 cells) (Table 1), namely abalone oligopeptide (AOP) and abalone anti-tumor peptide (AATP). AOP (Ala-Glu-Leu-Pro-Ser-Leu-Pro-Gly) was first characterized in 2013 by Nguyen et al. [90], while AATP (Lys-Val-Asp-Ala-Gln-Asp-Pro-Ser-Glu-Trp) was described in 2019 by Gong at al. [91]. In addition to inhibitory activity against MMPs, the AATP peptide also reduces the level of mRNA expression of both gelatinases.

Other peptides were also identified from boiled abalone such as ATPGDEG (Ala-Thr-Pro-Gly-Asp-Glu-Gly) and BABP (Glu-Met-Asp-Glu-Ala-Gln-Asp-Gly-Asp-Pro-Lys) (Table 1). In a human keratinocyte cell line (HaCaT cells), treatment with ATPGDEG resulted in a reduction of MMP-9 secretion. Moreover, molecular docking analysis suggested that ATPGDEG interacts with the MMP-9 active site, thereby blocking the catalytic activity [92].

The treatment of HT1080 cells with BABP also resulted in reduced levels of MMP-9 but not of MMP-2. BABP was able to suppress both MMP-9 protein levels (as determined by gelatin zymography and Western blot analysis) and mRNA expression in a dose-dependent manner [93].

Despite several studies, it is still not fully known how these peptides interact with MMPs. One hypothesis is that the amino acids Glu, Asp, Pro, and Lys could be involved [91].

The LSGYGP (Leu-Ser-Gly-Tyr-Gly-Pro), a peptide isolated from tilapia fish skin gelatin hydrolysates (TGHs) (Table 1), showed a high hydroxyl radical scavenging activity in an in vivo study. It has been suggested that TGHs could protect mouse skin collagen fibers against UV irradiation damage. In UVB-stimulated mouse embryonic fibroblasts (MEFs), LSGYGP significantly decreased the levels of MMP-9 in a dose-dependent manner. Furthermore, after using molecular modeling simulation, it was suggested that LSGYGP can enter into the catalytic site of MMP-9, thereby inhibiting its proteolytic activity [94]; however, in vitro or in vivo evidence of its effect on MMP-9 proteolytic activity is not yet available.

The Mere Meretrix 15 kDa polypeptide (Mere15) peptide was isolated from *Meretrix meretrix Linnaeus*, a mollusk used in traditional Chinese medicine as an anticancer molecule (Table 1). In human lung adenocarcinoma A549 cells, Mere15 downregulated the secretion of proteins and the expression of the mRNA of MMP-2 and MMP-9. Moreover, MMP-9 expression was lower than that of MMP-2, suggesting that MMP-9 is more sensitive to Mere15 inhibition [95].

### 2.2. Compounds

Diazepinomicin (BU-4664L) is a terpenoid firstly identified in the extract of marine *Micromonospora* sp [96]. BU-4664L has mainly been studied for its anti-invasive and anti-migratory effects on cancer cells. In murine colon 26-L5 carcinoma cells, BU-4664L inhibited the proteolytic activities of MMP-2 and MMP-9 with IC_50_ values of 0.46 and 0.60 µg/mL, respectively (Table 2). Furthermore, it was confirmed that BU-4664L is able to influence both extracellular matrix degradation and cell migration [97].

In 2008, a Phase II clinical trial with BU-4664L was started in patients affected by glioblastoma. Unfortunately, due to a lack of efficacy, the trail failed. Nevertheless, BU-4664L is a bioactive, farnesylated, dibenzodiazepinone alkaloid that is able to cross the BBB and provide a potential scaffold for further optimization and for investigation in the study of the relationship between AD and MMPs.

Ageladine A, isolated from the extract of the marine sponge *Agelas nakamurai*, possesses antiangiogenic activity. An in vitro study on isolated enzymes showed that ageladine A inhibits both MMP-2 and MMP-9 proteolytic activity (Table 2), whereas the *N*-methylated derivatives did not inhibit MMP-9 [98]. Since several MMP inhibitors exhibit their inhibitory activity by interaction with the Zn^+2^-ion of the catalytic domain (chelation), the chelation power of ageladine A was investigated but could not be established. Therefore, ageladine A is likely to inhibit through a yet-to-be-determined mechanism [98].

Aeroplysinin-1 is a brominated antibiotic secreted by *Aplysina aerophoba* sponges as a chemical defense response triggered by tissue injury. Aeroplysinin-1 is thought to have anti-tumor and anti-angiogenic actions. A study conducted in different human endothelial cell lines reported a decrease of MMP-2 expression [99]; see Table 2. Interestingly, aqueous extracts of *Aplysina aerophoba* were able to reduce the protein and mRNA expression of both MMP-2 and MMP-9 in rat astrocyte cultures [101].

Lemnalol (Table 2), isolated from soft coral (*Lemnalia cervicornis* and *Lemnalia tenuis Verseveldt*), has anti-inflammatory effects on mast cells (MCs) and osteoclasts activity in rats with monosodium urate (MSU) crystal-induced gouty arthritis. Though immunohistochemical analysis, it was shown that lemnalol decreases the infiltration and degranulation of MCs, and it was suggested that this effect is partially related to reduced expression of MMP-9 [100].

11-*Epi*-sinularoide acetate (11-*epi*-SA) was isolated from the soft coral *Sinularia flexibilis* (Table 2). In hepatocellular carcinoma cells (HA22T cells), 11-*epi*-SA was found to inhibit cell migration and invasion in a concentration-dependent manner. These activities remained at low, non-toxic doses (<7.98 μM) and also reduced the expression and activity of MMP-2 and MMP-9, suggesting that the effect could be associated with the modulation of MMPs or their endogenous inhibitors [102].

Recently, 11-*epi*-SA was also studied in a human bladder cancer cell line (TSGH-8301 cells), again showing a relevant effect against cell migration and invasion. Similarly, these effects were associated with decreased levels of MMP-2 and MMP-9 protein secretion [103]. Considering the reported results in cancer models, 11-*epi*-SA might also be a promising candidate for further development as a new modulator of MMPs in other pathologies such as AD.

Dihydroaustrasulfone alcohol (DA), isolated from marine coral, has antioxidant and anti-cancer activity (Table 2). Furthermore, DA has a concentration-dependent inhibitory effect on the migration and motility of human non-small cell lung carcinoma cells (NSCLC A549 cells), as determined by trans-well and wound healing assays. Gelatin zymography analysis, a standard method to detect MMP-2 and MMP-9 levels and proteoforms, showed that DA also significantly inhibited the presence of MMP-2 and MMP-9. These results proved that the anti-metastatic effect of DA was associated with the suppression of enzymes involved in cancer cell migration [104].

DA has also been proposed as an anti-restenosis molecule. Restenosis is characterized by the abnormal proliferation and migration of vascular smooth muscle cells (VSMCs) and the stimulation of platelet-derived growth factor (PDGF)-BB. Based on gelatin zymography data, it was suggested that DA dose-dependently decreased the pro-forms of all gelatinases, as well as the active form of MMP-9, in comparison with the control group (PDGF-BB alone). These results showed that DA decreased the activation and expression levels of both MMP-2 and MMP-9, which are involved in cell migration [105].

## 3. Natural Products from Terrestrial Source That Modulate MMP-2 and/or MMP-9

Natural bioactive products, phytochemicals, and nutraceuticals extracted from plants, minerals, animals, and microorganism have been the source of most of the bioactive molecules used in traditional medicine. Several in vitro and in vivo studies have demonstrated the therapeutic potential of natural products in various pathologies including degenerative and neurodegenerative diseases such as AD, PD, HD, ALS, and MS [21,106,107,108,109,110,111].

**Figure 6:**
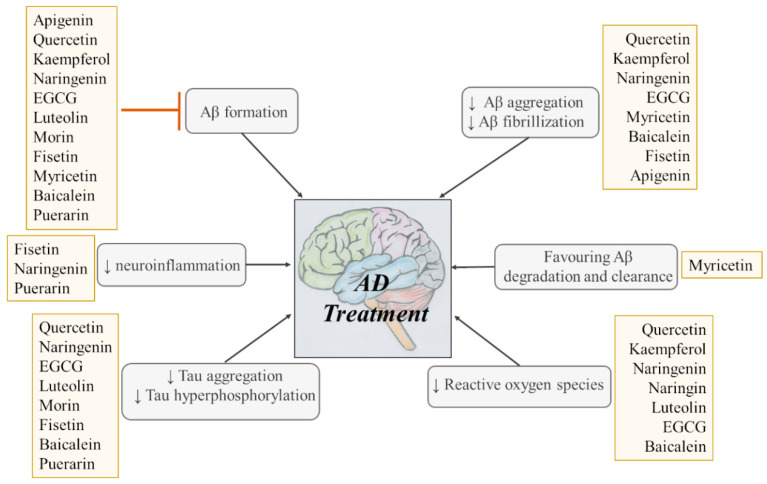
Overview of flavonoids with modulatory activity on MMP-2/-9 and with confirmed effects on different processes involved in Alzheimer’s disease (AD) pathology. EGCG: epigallocatechin gallate.

Due to their multifunctional properties, natural products may interfere with AD progression at all stages including the formation and clearance of pathological aggregates, the release of damaging reactive oxygen species, and even neuroinflammation. Recently, Andrade et al. generated a detailed overview of the state of the art of natural molecules and natural extracts currently studied in AD [21]. Interesting, the majority of natural molecules reported as a potential drug candidate against AD also influence MMP-2 and MMP-9. In Figure 6, an overview is given of flavonoids with MMP-2/-9 modulatory activity, including their confirmed effects on pathological processes in AD. Below, we further discuss the effects of these flavonoid on MMP-2 and MMP-9 mRNA expression, protein production, and proteolytic activity, as reported in several different disease models, in vitro cell-based assays, and biochemical assays.

### Flavonoids

Flavonoids are phenolic compounds that can be isolated from a wide range of plants. Several of these compounds have been attributed beneficial actions in health and disease. Their main effects include anti-carcinogenic, anti-inflammatory, antiviral, antioxidant, and psychostimulant activities. In the following section, we report on flavonoids with known positive effects in AD [21] and that have the ability to module MMP-2 and MMP-9; see Table 3.

Quercetin (Que), Table 3, has been largely studied for its anti-inflammatory and anti-cancer activity.

In the human fibrosarcoma cells line (HT1080), Que was found to inhibit both MMP-2 and MMP-9 protein levels were in a dose-dependent manner [112]. In a human hepatocarcinoma cell line (HCCLM3 cells), Que inhibits cell migration and invasion in vitro, and it has been suggested that these anti-migratory and anti-invasive effects are due to the ability of Que to downregulate the protein expression of MMP-2 and MMP-9 [113]. Moreover, the effect of Que was also studied in human oral cancer cell lines (HSC-6 and SCC-9 cells), where Que also decreased the abundances of MMP-9 and MMP-2 [114].

In an asphyxia-based rat model for cardiopulmonary resuscitation (CPR), rats treated with treated by intragastric injection of 50 mg/kg quercetin once a day for five days had significantly less reactive oxygen species (ROS) generation, inflammation, and MMP-2 protein expression [147]. Que was also studied in the two-kidney one-clip rat model for hypertension. Animals treated with quercetin (10 mg/kg/day for three weeks by gavage) had reduced aortic ROS levels and MMP-2 activity, as determined by situ zymography and immunofluorescence [115].

Kaempferol (Kae), Table 3, is a bioactive substance that possesses several properties such as anti-cancer, anti-diabetic, anti-inflammatory, anti-aging, anti-allergic, and cardio-protective activities [148]. In human tongue squamous cell carcinoma cells (SCC4 cells), Kae inhibited migration and invasion, reduced the protein expression of MMP-2, and decreased the nuclear translocation of the transcription factor AP-1 to the MMP-2 promoter [116].

As a phytoestrogen, Kae is known to play a chemopreventive role inhibiting carcinogenesis and cancer progression. In the MCF-7 breast cancer cell line, Kae inhibits the protein expression of epithelial-mesenchymal transition-related markers and suppresses metastasis-related markers such as MMP-2 and MMP-9 [117].

Naringenin (Nar), Table 3, is a bioactive compound found in several fruits that has anti-inflammatory and antitumor effects. One study investigated the effect of Nar on the migration of lung cancer cells (A549 cells) and found a significant alteration in A549 cell proliferation in response to Nar treatment. Gelatin zymography revealed that Nar reduces MMP-2 and MMP-9 levels in a concentration-dependent manner [118].

Epigallocatechin Gallate (EGCG), Table 3, is the most abundant catechin found in green tea. EGCG has various biological effects such as antioxidant, radical scavenging, antimicrobial, anti-inflammatory, anticarcinogenic, antiapoptotic, and metal-chelating activities [149]. Moreover, several studies have reported that EGCG offers potential protection from neurodegeneration [150] or can be considered an inhibitor of cancer cell metastasis via the inhibition of the expression and activity of several proteins such as MMP-2 and MMP-9. MCF-7 cells treated with EGCG suppress the expression of pro-MMP-2 [119]. In a human breast cancer cell line (MDA-MB-231) with high metastatic properties, treatment with EGCG resulted in the inhibition of MMP-9 mRNA and protein expression [120]. Additionally in human pancreatic cancer cells (AsPC-1 cells), EGCG inhibits the expression of MMP-2 and MMP-9 [121]. Furthermore, in biochemical assays with recombinant MMP-9, the direct inhibition of MMP-9 gelatinolytic activity by EGCG has also been established [32].

Luteolin (Lut), Table 3, is largely present in herbs, vegetables, and fruits, and it exhibits anti-inflammatory and antioxidant activities. The effect of Lut on MMP-2 and MMP-9 in azoxymethane (AOM)-induced colon carcinogenesis in BALB/c mice was investigated. The expression of MMP-2 and MMP-9 was increased during AOM induction, whereas treatment with Lut (15 mg/kg, intraperitoneally, once a week for three weeks) reduced their expressions reduced their expressions [122]. Recently, a study showed that Lut inhibits the metastasis of ovarian cancer cells (A2780 cells) by downregulating the expression of MMP-2 and MMP-9 both in vitro (A2780 cells) and in vivo in a tumor model comprising subcutaneous injection of A2780 cells in nude mice [123]. Lut exhibited a similar behavior in human melanoma cells (A375 cells), where it inhibits proliferation, induces apoptosis, and reduces the expression of MMP-2 and MMP-9 (in vitro and in vivo) [124].

Morin (Mor), Table 3, is a flavonol with various bioactive properties including neuroprotection, the suppression of inflammation, and anticancer activity [151]. An in vivo study was performed to evaluate the role of Mor in diethylnitrosamine (DEN)-induced hepatocarcinogenesis in Wistar albino rats. Both MMP-2 and MMP-9 levels were increased in DEN-induced animals when compared to a control. In animals treated with Mor, MMP-2, and MMP-9 levels were decreased [125]. Next, Mor was also tested in cultured LX-2 cells (hepatic stellate cells; HSCs) and diethylnitrosamine-induced fibrotic rats. Again, significantly decreased levels of MMP-2 and MMP-9 were found upon Mor treatment (200 mg/kg in drinking water) when compared to untreated cells and DEN-induced fibrotic rats [126].

Recently, Mor hydrate was studied in the metastasis of MCF-7 human breast cancer cells, where Mor hydrate suppressed 12-*O*-tetradecanoylphorbol-13-acetate (TPA)-induced cell migration and invasion via the inhibition of MMP-9 mRNA and protein expression [127].

Apigenin (Api), Table 3, a flavonoid present in vegetables, fruits, and herbs, possesses several bioactive properties, including anti-inflammatory, neuroprotective [152], and anticancer activities [153].

In colorectal adenocarcinoma cell lines (SW480 cells), Api reduced MMP-9 protein levels in a dose-dependent manner correlating with anti-metastasis and antitumor effects [128].

The positive action of Api was also evaluated in U87 glioma cells, where it was found to reduce tumor cell metastasis and invasion, inhibit MMP-9 mRNA levels, and downregulate nuclear factor-_K_B (NF-_K_B) [129], a known regulator of MMP-9 expression under inflammatory conditions. The anti-metastatic action of Api was also found in human melanoma cells (A375 cells), where Api reduces the MMP-2 and MMP-9 in a dose-dependent manner [130].

Fisetin (Fis), Table 3, is a bioactive flavonol found in several fruits and vegetables, and it is recognized for its anti-inflammatory, anti-proliferative, and neuroprotective effects [154,155].

Fis is able to dose-dependently inhibit MMP-9 protein and mRNA expression in a pancreatic cancer cell line (AsPC-1 cells) [131]. Recently, the effect of Fis on MMP-2 and MMP-9 expression in triple breast cancer cells (4T1 and JC cells) was investigated, with the findings that Fis reduced cell motility and that this phenomenon was partially associated with a reduction of MMP-2 and MMP-9 expressions [132].

Myricetin, (Myr), Table 3, is abundantly found in vegetables, fruits, teas, and some medicinal plants. Several studies have illustrated that Mir can exert anti-oxidant, anti-inflammatory, anti-cancer, and neuroprotective effects [156]. Myr acts as an anti-cancer agent through different mechanisms including the modulation of MMP-2 and MMP-9. It inhibits MMP-2 protein expression in colorectal carcinoma cells (COLO 205). Furthermore, one study reported that purified MMP-2 incubated with Myr had reduced activity when analyzed by gelatin gel zymography [133], which would suggest a direct and strong Myr/MMP-2 interaction. Recently, it has been reported that Myr suppresses breast cancer metastasis through the downregulation of the activity of MMP-2 and MMP-9 (MDA-Mb-231Br cells) [134]. In another study, the effect of Myr on the migration and invasion of radioresistant lung cancer cells (A549-IR cells) was investigated. Experimental evidence showed that Myr can inhibit the invasion and migration of A549-IR cells by suppressing the expression of MMP-2 and MMP-9 through the inhibition of the focal adhesion kinase FAK-ERK signaling pathway [135].

Recently, Myr has been studied in a pentylenetetrazole (PTZ)-induced mouse model of epilepsy (C57BL/6 male mice). It is known that PTZ-induction increases the expression of MMP-9 and that the selective inhibition of MMP-9 confers neuroprotection in patients with epilepsy. Interestingly, treatment with Myr (100–200 mg/kg, orally for 26 days, 30 min prior to each PTZ injection) reduced the mRNA and protein levels of MMP-9 in a dose-dependent manner, thus confirming the neuroprotective role of Myr [136]. 

Baicalein (Bai), Table 3, is produced by the root of Chinese skullcap, *Scutellaria baicalensis* Georgi (Lamiaceae), and it is a bioactive substance part of traditional Chinese medicine. Bai has several beneficial effects conferred through its anti-oxidant, anti-viral, anti-inflammatory, anti-angiogenic, and anti-cancer activities [157]. Moreover, studies have shown that Bas exerts a neuroprotective role in AD [158].

Several studies have reported that Bai acts as an anticancer agent through several pathways including the modulation of MMP-2 and MMP-9 expression. In a benzo(a)pyrene-induced pulmonary carcinogenesis mouse model, animals treated with Bai (12 mg/kg) had the significantly reduced mRNA and protein expression of MMP-2 and MMP-9 [137].

The anti-proliferative potential of Bai was also studied in melanoma cell lines (A375 and SK-MEL-28), where MMP-2 expression was significantly reduced in cells treated with Bai [138]. Moreover, Bai was tested in a pancreatic neuroendocrine tumor cell line (BON1 cells), and the observed reduction of tumor migration and invasion related to the decreased MMP-2 and MMP-9 [139]. Recently, it has been reported that Bai contributes to reduced metastasis in osteosarcoma. Upon treatment with Bai, the invasive capacity of human osteosarcoma cells (CRL-1427 cells) was reduced. This result was attributed to a series of enzymes modulated by Bai, including the reduced production of MMP-9 and MMP-2 [140].

Puerarin (Pue), Table 3, is part of the isoflavone glycoside group, and it is extracted from *Pueraria lobate*, *Pueraria thomsonii*, and *Pueraria tuberosa*. Pue was approved by the Chinese Ministry of Health for clinical treatment in 1993. It was primarily used for the treatment of cardiovascular diseases and later also reported to have anticancer activity [159]. Recently, studies have reported the ability of Pue to protect against AD [160]. Pue is known for its properties including bone-sparing, anti-inflammatory, and anti-proteinase properties. Rats with periodontitis that were treated with Pue showed a reduction of MMP-2 and MMP-9 expression [141]. Pue also significantly inhibited lipopolysaccharide (LPS)-induced MCF-7 and MDA-MB-231 cell migration, invasion, and adhesion. The mRNA and protein levels showed that Pue treatment effectively negated the expression of several proteins including MMP-2 and MMP-9 in LPS-activated cells [142].

Rutin, (Rut), Table 3, is a polyphenolic natural flavonoid known as quercetin-3-*O*-rutinoside and vitamin P, that is found in vegetables, citrus fruits, and plant-derived beverages. Rut has been largely studied for its several bioactive properties [161]. Rut was studied in vivo in a rat photothrombotic cerebral ischemic model, and the administration of Rut reduced BBB disruption via the downregulation of MMP-9 protein expression [143]. In mice with LPS-induced heart injury, Rut mitigated fibrosis-related genes, reduced MMP-2 and MMP-9 levels in the heart, and prevented LPS-induced cardiac fibrosis [144].

Naringin (Nar), Table 3, is a bioflavonoid compound especially found in grapefruit and is related to citrus herb species. It has an extensive spectrum of pharmacological activities such as anti-inflammatory, anti-oxidant, antitumor, and neuroprotective effects [162].

In a glioma cell line (U251 cells), Nar inhibited invasion and migration at several concentrations. In addition, a decrease in the levels of MMP-2 and MMP-9 was measured [145]. Similar results were obtained in human glioblastoma cells (U87 cells), where Nar exhibited inhibitory effects on the invasion and adhesion of U87 cells and reduced the protein levels of both MMP-2 and MMP-9 [146].

## 4. Conclusions and Future Prospective

AD is a chronic crippling disease for which the approved drugs are only palliative. In this manuscript, we briefly discuss the complex role that MMPs, specifically the gelatinases MMP-2 and MMP-9, have in neurodegeneration and neuroinflammation. It is clear that by modifying structural proteins and altering the functions of cytokines, MMPs contribute to the progression of neuropathology. In addition, in several in vivo studies, the beneficial effects of targeting MMPs in neuroinflammation and neurodegeneration were confirmed.

Both in clinical and preclinical studies, it was shown that many natural products from marine and terrestrial sources are promising bioactive substances for AD treatment. Interestingly, many of these natural products also have the ability to modulate MMP-2 and MMP-9. In this manuscript, we summarized the MMP-2 and MMP-9-modulatory activities of marine and terrestrial compounds with known beneficial effects on processes involved in AD pathology. Many natural compounds appear to regulate signal transduction pathways, thus leading to the simultaneous downregulation of MMP-2 and MMP-9 gene and protein expression. While such effects might correspond with general anti-oxidant and anti-inflammatory properties, for some compounds, direct anti-proteolytic activity on MMP-2 and/or MMP-9 has been established (e.g., ATPGDEG, LSGYGP, BU-4664L, ageladine A, quercetin and myricetin). In addition, several compounds require further research to decipher their exact regulator mechanisms. For example, it is imperative to determine whether their activity is due to the direct inhibition of the proteolytic mechanisms of MMPs or due to the direct or indirect modulation of signaling pathways upstream of MMP production.

In conclusion, natural compounds might represent a pipeline or ‘blueprint’ to develop new molecules that can modulate MMPs. Furthermore, the inhibition of MMPs in combination with other properties such as anti-inflammatory and anti-oxidant activity, as well as abilities such as diffusion across the blood–brain barrier, might provide valuable for treatments against AD progression.

## Figures and Tables

**Table 1:** Peptides inhibitors of MMP-2 and MMP-9 from marine source.

Compound	Source	Sequence	MMP-2	MMP-9	Model	Ref.
**AOP**	*Haliotis discus hannai*	Ala-Glu-Leu-Pro-Ser-Leu-Pro-Gly	Inhibition ^2^	Inhibition ^2^	HT1080 cells	[90]
**AATP**	*Haliotis discus hannai*	Lys-Val-Asp-Ala-Gln-Asp-Pro-Ser-Glu-Trp	Inhibition ^2,3^	Inhibition ^2,3^	HT1080 cells	[91]
**ATPGDEG**	*Haliotis discus hannai*	Ala-Thr-Pro-Gly-Asp-Glu-Gly	n.d.	Inhibition ^1,2^	HaCaT cells	[92]
**BABP**	*Haliotis discus hannai*	Glu-Met-Asp-Glu-Ala-Gln-Asp-Gly-Asp-Pro-Lys	Inhibition ^2,3^	Inhibition ^2,3^	HT1080 cells	[93]
**LSGYGP**	*Tilapia fish skin gelatin hydrolysate*	Leu-Ser-Gly-Tyr-Gly-Pro	n.d.	Inhibition ^1,2^	mice	[94]
**Mere15**	*Meretrix meretrix Linnaeus*	unknown	Inhibition ^2,3^	Inhibition ^2,3^	A549 cells	[95]

AOP: abalone oligopeptide; AATP: abalone anti-tumor peptide; ATPGDEG: Ala-Thr-Pro-Gly-Asp-Glu-Gly; BABP: boiled abalone byproduct peptide; LSGYGP: Leu-Ser-Gly-Tyr-Gly-Pro; Mere15: Mere Meretrix 15 kDa polypeptide. ^1^ Inhibition of proteolytic activity (e.g., substrate degradation assays and molecular docking); ^2^ inhibition of protein expression (e.g., in gel zymography, Western-blot, and ELISA); ^3^ inhibition of mRNA expression (e.g., reverse transcription polymerase chain reaction, RT-PCR). n.d.: not defined.

**Table 2:** Compound inhibitors of MMP-2 and MMP-9 from marine sources.

Structure	Source	MMP-2	MMP-9	Model	Ref
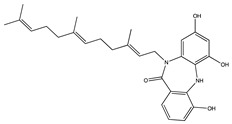 BU-4664L (Diazepinomicin)	*Micromonospora* sp.	Inhibition ^1^	Inhibition ^1^	26-L5 cells	[97]
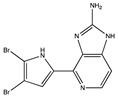 Ageladine A	*Agelas nakamurai*	Inhibition ^1^	Inhibition ^1^	Isolated enzymes	[98]
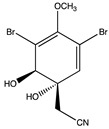 Aeroplysinin-1	*Aplysina aerophoba*	Inhibition ^2^	n.d.	Endothelial cells	[99]
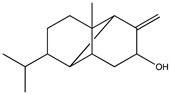 Lemnalol	*Lemnalia* sp.	n.d.	Inhibition ^2^	rats	[100]
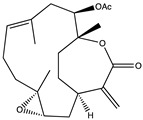 11-epi-sinulariolide acetate	*Sinularia flexibilis*	Inhibition ^2^	Inhibition ^2^	HA22T cells	[101] [102]
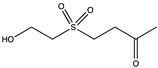 Dihydroaustrasulfone alcohol	*Cladiella australis*	Inhibition ^2^ Inhibition ^2^	Inhibition ^2^ Inhibition ^2^	A549 cells VSMC (vascular smooth muscle cells)	[103] [104]

^1^ Inhibition of proteolytic activity (e.g., substrate degradation assays, and molecular docking); ^2^ inhibition of protein expression (e.g., in gel zymography, Western blot, ELISA, and immunohistochemistry); n.d.: not defined.

**Table 3:** Flavonoid compounds able to modulate MMP-2 and MMP-9 levels.

Structure	Source	MMP-2	MMP-9	Model	Ref
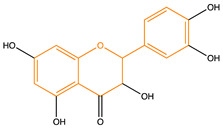 **Quercetin**	Fruit, vegetables, seeds, and grains	Inhibition ^1,2^	Inhibition ^2^	HT1080 cells HCCLM3 cells HSC-6 cells SCC-9 cells 2K1C rats	[112] [113] [114] [114] [115]
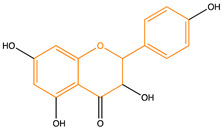 **Kaempferol**	Tea, cabbage broccoli, kale, beans, endive, tomato, strawberries grapes, and endive	Inhibition ^2^ Inhibition ^2^	n.d. Inhibition ^2^	SCC-4 cells MCF-7	[116] [117]
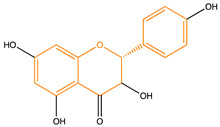 **Naringenin**	Grapefruit, bergamot, orange, tomatoes, and cocoa	Inhibition ^2^	Inhibition ^2^	A549 cells	[118]
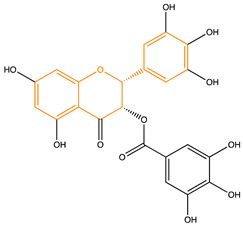 **Epigallocatechin Gallate**	Green tea	n.d. Inhibition ^2^ n.d. Inhibition ^2^	Inhibition ^1^ n.d. Inhibition ^2,3^ Inhibition ^2^	Biochemical assay MCF-7 cells MDA-MB-231 AsPC-1 cells	[32] [119] [120] [121]
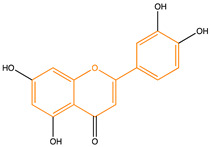 **Luteolin**	Salvia, broccoli, parsley, thyme, green pepper, and artichoke	Inhibition ^2^ Inhibition ^2^ Inhibition ^2^ Inhibition ^2,3^ Inhibition ^2^	Inhibition ^2^ Inhibition ^2^ Inhibition ^2^ Inhibition ^2,3^ Inhibition ^2^	mice A2780 cells mice A375 cells mice	[122] [123] [123] [124] [124]
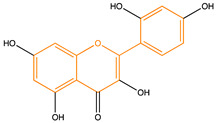 **Morin**	Osage orange, common guava, and old fustic	Inhibition ^2^ Inhibition ^2^ Inhibition ^2^ n.d.	Inhibition ^2^ Inhibition ^2^ Inhibition ^2^ Inhibition ^2,3^	rats LX-2 cells rats MCF-7	[125] [126] [126] [127]
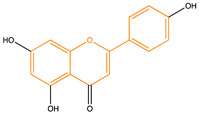 **Apigenin**	Chamomile grapefruit, parsley, celery, celeriac, and onion	n.d. n.d. Inhibition ^2,3^	Inhibition ^2^ Inhibition ^3^ Inhibition ^2^	SW480 cells U87 A375 cells	[128] [129] [130]
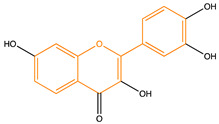 **Fisetin**	Kiwifruit, tomato, strawberries, apples, persimmons, onions, and cucumbers	n.d. Inhibition ^2^ Inhibition ^2^	Inhibition ^2,3^ Inhibition ^2^ Inhibition ^2^	AsPC-1 cells 4T1 cells JC cells	[131] [132] [132]
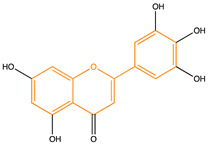 **Myricetin**	Tomatoes, oranges, nuts, berries, tea, and red wine	Inhibition ^1,2^ Inhibition ^1,2^ Inhibition ^2^ Inhibition ^2,3^ Inhibition ^2,3^	n.d. n.d. Inhibition ^2^ Inhibition ^2,3^ Inhibition ^2,3^	Isolated MMP-2 COLO 205 cells MDA-Mb-231Br cells A549-IR C57BL/6 mice	[133] [133] [134] [135] [136]
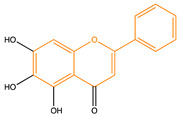 **Baicalein**	Root of *Scutellaria baicalensis*	Inhibition ^2,3^ Inhibition ^2^ Inhibition ^2^ Inhibition ^2^ Inhibition ^2^	Inhibition ^2,3^ n.d. n.d. Inhibition ^2^ Inhibition ^2^	mice A375 SK-MEL-28 BON1 cells CRL-1427 cells	[137] [138] [138] [139] [140]
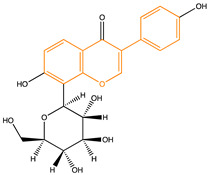 **Puerarin**	Root of *Pueraria thomsonii*, *Pueraria tuberosa*, and *Pueraria lobate*	Inhibition ^2^ Inhibition ^2,3^ Inhibition ^2,3^	Inhibition ^2^ Inhibition ^2,3^ Inhibition ^2,3^	rats MCF-7 cells MDA-MB-231 cells	[141] [142] [142]
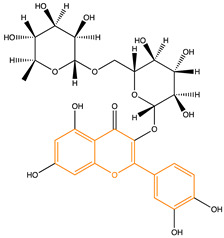 **Rutin**	Capes, olive buckwheat, asparagus, red- raspberry, tomato, prune, fenugreek, zucchini, and apricot	n.d. Inhibition ^2^	Inhibition ^2^ Inhibition ^2^	rats rats	[143] [144]
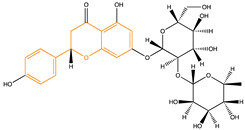 **Naringin**	citrus fruits, grapefruit, artichokes, brussels sprouts, strawberries, rosemary, oregano, and tomato	Inhibition ^2^ Inhibition ^2^	Inhibition ^2^ Inhibition ^2^	U251 cells U87 cells	[145] [146]

Flavonoid scaffold is highlighted in orange. ^1^ Inhibition of proteolytic activity (e.g., substrate degradation assays, molecular docking, and in situ zymography); ^2^ inhibition of protein expression (e.g., in gel zymography, Western blot, ELISA, and immunohistochemistry); ^3^ inhibition of mRNA expression (e.g., RT-PCR). n.d.: not defined.
